# Progress in Teaching Physician–Patient Communication in Medical School; Personal Observations and Experience of a Medical Educator

**DOI:** 10.5041/RMMJ.10037

**Published:** 2011-04-30

**Authors:** Shimon Glick

**Affiliations:** Moshe Prywes Center for Medical Education, Faculty of Health Sciences, Ben Gurion University of the Negev, Beer-Sheva, Israel

**Keywords:** Physician-patient communication, medical education, communication skills, medical school curriculum

## Abstract

In spite of the enormous progress of Western medicine during the past century there has not be a concomitant rise in the public’s satisfaction with the medical profession. Much of the discontent relates to problems in physician–patient communication. The multiple advantages of good communication have been clearly demonstrated by numerous careful studies. While the past few decades have witnessed much more attention given to teaching communication skills in medical schools, there are a number of factors that create new problems in physician–patient communication and counteract the positive teaching efforts. The “hidden curriculum”, the increased emphasis on technology, the greater time pressures, and the introduction of the computer in the interface between physician and patient present new challenges for the teaching of physician-patient communication.

There has been a proliferation of literature on the teaching of physician-patient communication in medical school during the past several decades. 1–5 The present article will not attempt to reduplicate the existing reviews of the subject, which are readily available in the accessible medical literature.

Instead, as one who began his medical studies some 60 years ago and has been associated with academic medical institutions on two continents for several decades, I will present some personal impressions as part of what might be termed “narrative medicine”. Hopefully some of these experiences and suggestions will prove useful to the readers of the journal.

## PARADOX OF SOCIETAL DISSATISFACTION WITH PHYSICIANS

There exists a troubling paradox in the field of Western medicine. The progress of medicine in the past century has been almost miraculous. The understanding of disease processes down to the molecular level has progressed daily; specific treatments have been found for dozens of formerly untreatable diseases. Paul Beeson at his retirement from editorship of the Cecil and Loeb classic text-book of medicine noted^6^ that in the 38 years of his editorship the number of diseases for which there had been specific therapy increased from what had been 5%–10% to 50%–55%. The past half-century has witnessed the introduction of organ transplantation, open heart surgery, renal dialysis, cure of some cancers, *in-vitro* fertilization, and many other advances. One would have expected that the public admiration of the physician would have increased as dramatically as have the advances in medicine. Yet in spite of these remarkable contributions of medicine to the health and welfare of the public, there may actually be increased rather than decreased dissatisfaction of the public with their physicians. To quote a recent New York Times article: “a growing chorus of discontent suggest that the once revered doctor–patient relationship is on the rocks” 7 A Time magazine cover in 1989 showed the symbol of the physician as a poisonous snake, rather than as a healing Aesculapian serpent, with the heading “image versus reality”.^8^ The increasing incidence of malpractice suits and the growing use of alternative/complementary medicine are all clear indications of the public’s dissatisfaction with the care they are getting from their physicians. Almost all analyses of the perceived problems with physicians relate not to the scientific defects of the physician but to problems in physician–patient communication.^9^

## HISTORICAL DEFICIENCIES IN TEACHING COMMUNICATION SKILLS

When I attended medical school over half a century ago there were no courses whatsoever on physician–patient communication. It is almost amusing to recall the *only* formal discussion that I remember from my student days. One of the senior attending physicians in the department of obstetrics and gynecology told us on rounds one morning that if the patient requests information about her disease the most useful word to use is “condition”. “Just tell her”, he said, “that she has a ‘uterine condition’, without any further elaboration. That will satisfy 95% of the patients, and you will not have to supply any further details about her diagnosis.” That was the sum of the teaching of communication skills that was provided to me and my fellow students during 4 years of medical school! Nor were these defects remedied in any significant way during my residency training at outstanding academic institutions. But the lack of attention to communication skills has come to haunt the medical profession. Perhaps the research published in the late 1960s by Barbara Korsch and her colleagues,^10^–^11^ highlighting the gaps in doctor–patient communication, provided the scientific impetus for further research and then remediation of the situation. Recent articles continue to report serious shortcomings in communication skills.^12^ Even with outstanding formal teaching unless there is reinforcement during the clinical years and good role models the gains of the teaching may deteriorate significantly under the stresses of work and the “hidden curriculum”.

## “PATHOPHYSIOLOGY” OF DEFICIENCIES IN PHYSICIAN–PATIENT COMMUNICATION

One should examine first the “pathophysiology” of the problem in physician–patient communication in order to prescribe appropriate solutions.

## PRIORITIES OF TECHNOLOGY

The advent of technological and sophisticated methods of diagnosis and treatment of disease has relegated the communicative interaction with the patient to a lower priority. A leading daily newspaper pointed out the following: “The CT and MRI scans, the lasers and the laparoscopics, the chemo cocktails and DNA codes – all the advances that make modern medicine so effective (and expensive) have isolated physicians from the patient as a person. In the process, the ancient therapeutic art of listening is being ignored, much to the dismay of many physicians who recognize the limits of technology.”^13^ Going back to the invention of Laennec, who introduced the stethoscope to replace the direct placement of the physician’s ear on the patient’s chest, we have progressively decreased the direct contact of the physician with the patient. I still recall during my internship watching the great clinician cardiologist William Dressier carefully percussing the precordium in order to diagnose a pericardial effusion, a unique skill which he so carefully demonstrated for us. But this anachronistic expertise has been almost totally replaced by the simple and much more reliable ultrasound.

An uncle of mine died with pneumococcal pneumonia in the pre-antibiotic era. The family physician sat by the bedside repeatedly over several days waiting for the “crisis” which signaled recovery or for death. But several injections of penicillin turned out to be infinitely more effective than a physician’s empathic care.

The impressive success of technology has simply pushed the classic skill of communication into a seemingly minor role in patient care. In addition, the reward system which willingly pays much more for a simple manual procedure than for cognitive and interpersonal activities, delivers a similar message. And the patient population confirms this set of priorities. When presented with a large bill for cognitive services one may hear: “But he did nothing; he just spoke to me.”

## SPECIALIZATION OF MEDICINE

Another major factor in the downgrading of communication skills has been the specialization and sub-specialization that has brought many benefits and sophistication to patient care. But this fragmentation of patient care has minimized long-term relationships with patients and the inclination of the practicing physician to take a holistic approach to the patient rather than focusing on his/her area of specific expertise.

## SOCIETAL CHANGES

There have also been major societal changes in the past few decades, with less emphasis on social responsibility and much greater tendency for individualism and self-fulfillment. This societal change has not bypassed the physicians, perhaps making them less empathic and sensitive to the needs of others.

## “HIDDEN CURRICULUM”

But even when interpersonal skills are taught in one form or another in the formal curriculum of the medical school, these attitudes are often eroded by what has been termed the “hidden curriculum”.^14^ The harassed and stressed surgical resident on night duty with the student often may deride the values and skills emphasized formally.

## SOCIETAL DISSATISFACTION

Over the past few decades the medical profession has found itself faced by rising numbers of malpractice suits and by the massive growth of use of complementary and alternative medicine.

Virtually all studies that have been done to examine which physicians are prone to being sued for malpractice have come to the same conclusion. Perhaps the major factor is a failure in physician–patient communication. Careful studies have shown that a physician’s style of communication is either likely to encourage malpractice suits or will protect against the likelihood of malpractice suits.^9^,^15^ Even the voice of the physician may be a factor.^16^ More and more group practices of physicians have begun to place greater emphasis on communication skills in hiring and retaining physicians, if only for economic reasons.

## PATIENT-CENTERED MEDICINE

Another factor in the increasing emphasis on communication skills are the societal changes in which patients now insist on much greater involvement in their health care decisions. This has been called an evolution from physician-centered medicine to patient-centered medicine.^17^ Even before the wide-spread use of the Internet, patients have become more assertive, demanding as a “right” the provision of more detailed information, and they insist on a greater role in deciding on specifics of diagnosis and treatment. The civil rights movement has had its parallel in the growth of the concept of patients’ rights, a concept that did not really achieve mainstream acceptance until the 1970s.

Not all physicians welcomed these changes. Some felt threatened by the concept that patients might question their advice. But in essence they have had no choice. A societal revolution has enveloped them, and, willingly or not, the world had changed.

## ADVANTAGES OF GOOD COMMUNICATION

In my days at medical school they talked about “bedside manner”, a vague term which suggested some degree of “professional” behavior intended to impress the patient with the physician’s stature and skills. These behaviors were neither defined nor taught.

Yet the value of empathic communication with patients was not all that new; it had been discussed impressively many years earlier. William Peabody’s classic article “The care of the patient” published almost a century ago^18^ was distributed to thousands of entering medical students throughout the United States over many decades. Its classic admonitions retain eternal validity. And George Engel’s call^19^,^20^ for a move to a bio-psycho-social approach to medicine was along similar lines. But serious research on the impact of good physician communication began to flourish only years later.

The serious deficiencies in physician communication skills were highlighted by Korsch and her colleagues^10^–^11^ and by numerous subsequent researchers.^12^–^21^ There is now a clear consensus among medical educators^1^–^5^ that formal teaching programs in communication skills should be an essential part of the medical curriculum. Much research has shown unequivocally that the old cliché about the advantages of “good bedside manner” does not represent some luxury but that good communication has multiple tangible benefits for patient, physician, and society. That patients felt better if the physician communicated well was not surprising. But many other tangible benefits have since been described. It turned out that physicians who communicate well feel better and suffer less “burn-out”.^22^ Physicians who communicate well elicit not only more information about psychosocial issues, as might be expected, but are rewarded by more important biomedical information as well,^23^ because a trusting patient will be much more open and revealing. But not only will more information be elicited, but patient compliance will be increased,^22^ and the objective patient response to therapy will be enhanced.^24^–^26^ Finally, using “bottom-line” inducements, the costs for malpractice suits will be decreased.^9^,^15^

## OBJECTIVES OF TEACHING COMMUNICATION SKILLS

As a result of all these findings medical schools began to teach communication skills several decades ago, and considerable experience has accumulated over these decades. One of the first reports published under the rubric *Medical School Objectives Project* by the Association of American Medical Colleges (AAMC) was a report on communication in medicine.^27^ Both the American Liaison Committee on Medical Education and the Canadian Council for the Accreditation of Canadian Medical Schools require formal teaching of communication skills in the curriculum. Similarly the Accreditation Council for Graduate Medical Education has placed specific emphasis on the teaching and evaluation of communication skills in all approved residency programs. Detailed curricula are readily available from many different sources,^28^–^30^ and I will not present specific data but rather some general observations.

As in other areas of medical education one needs to address the triad of knowledge, attitudes, and skills. The primary emphasis in communication teaching should obviously be on skills to be developed, but attention must also be paid to the provision of an adequate knowledge base and to the insistence on appropriate attitudes on the part of the student.

## TIMING OF TEACHING COMMUNICATION SKILLS

When should teaching of communication skills take place? Many will argue seemingly logically that when the students start to prepare for their clinical clerkships they should get training in communication skills. My own strong prejudice is that the appropriate time to start is at the very beginning of medical school. There have been several studies depicting the socialization changes that medical students undergo during their studies.^31^–^33^ As the dean in the Patch Adams movie tells the entering students, “We will change you from human beings into physicians”. The students are most receptive to learning about communication during their early years, when they still identify with the patients before they begin to identify more and more with the members of their profession. At this early stage they do not yet know very much about diseases, and when they speak to a patient they can discuss with them mostly about what diseases do to them as human beings. In the beginning of their clerkships they are appropriately more concerned about learning physical diagnosis, pathophysiology, pharmacology, etc., and teaching them detailed communication skills may be regarded as burdensome and fall upon partially deaf ears. Sadly studies have shown serious fall-offs in empathy as students move through medical school.^34^,^35^ Thus I believe that the early days of medical school when they are still ordinary “mortals” is the best time for “imprinting”, to teach them how illness impacts upon a human being and how to communicate with a patient.

During the training in the first two years of medical school the AAMC document^27^ proposes three goals: 1) The development of an appreciation of the interpersonal and situational dynamics of medical encounters. 2) The becoming oriented to the communication tasks of a physician. 3) The beginning to build a base of skills and strategies associated with these tasks.

But it is essential that teaching communication skills not be confined to the preclinical years. In subsequent years there should be both enforcement of the material learnt earlier as well as instruction in dealing with more difficult and challenging communication problems, such dealing with the dying patient, the angry patient, and delivering bad news. These are best taught in the related clinical settings. Thus such subjects as dealing with breaking bad news and the handling of difficult situations might well be part of the teaching in oncology, in the intensive care unit, or in the premature nursery. It is also essential to prevent the erosion of the attitudes and skills achieved in the early years by contacts with many role models who have not the appropriate attitudes and skills, yet have an active teaching role and influence.

## SPECIAL PATIENT GROUPS

In recent years increasing attention is being paid to cultural competences^36^ In our globalization era physicians will be called upon frequently to deal with patients from cultures vastly different than their own. Ann Fadiman’s book *The Spirit Catches You and You Fall Down37* is a dramatic description of the tragic failure of communication between conscientious physicians in dealing with illness in a family whose values were foreign and not understood by the health professionals. Physicians must learn how to deal effectively with different cultures.

It is important too that physicians learn how to communicate with patients who are handicapped by blindness, deafness, mental retardation, and psychiatric problems. We have had remarkably favorable feedback from our students by a weeklong exposure to such individuals during the students’ clinical week in the first year of medical school. 38

## QUALIFICATION OF TEACHERS

The question of who shall do the teaching is often a critical one. Unquestionably professionals in the behavioral sciences, particularly the applied ones, have the specialized academic training in the field, rarely matched by most clinicians. However, for a whole variety of reasons, some good and some bad, it is critical to have practicing physicians in major roles in the teaching. They have practical experience, and medical students relate better to them than they do to non-physicians. The more senior the physicians who play an active role in teaching communication skills, the more likely it is for students to take the teaching seriously. When teaching communication skills is left to junior house staff a clearly negative message is conveyed as to the priority assigned to such teaching.

## TEACHING TECHNIQUES

There are a variety of different techniques that have been shown to be effective, and probably one should vary the techniques. These include small group discussions/seminars, student interviews with simulated patients, student observations of faculty with real patients, student interviews with real patients, role-playing with peers, rounds, video trigger tapes with discussion,^39^,^40^ videotapes of student-patient interactions, instructional videotapes, literature study, especially personal accounts about physician illness^41^–^43^ among other modalities. When students see their own real-life performance on videotape followed by a non-threatening and constructive analysis by classmates and instructors this process has a powerful impact.

Faculty development in the area of communication skills and teaching is essential, so that the messages of the teaching are not only not eroded but are reinforced all along the years in medical school.

In an era of managed care and increasing economic pressures on physicians, students will often point out that in real life one does not have enough time in the patient encounter to apply what they learn in the courses on communication. It is certainly true that were one to have a half hour for each encounter it would be much easier to deal more effectively with the more personal aspects of the patients’ problems. Nevertheless there are data which show that more depends on the skill of the physician than on the time available. A study in a large and busy emergency room in a pediatric hospital showed^10^ that mothers’ satisfaction with the physicians’ communication did not depend on the number of minutes spent with the patient. An experienced family physician has described the manner of proper and effective physician–patient communication even in a 5–7-minute patient encounter.^44^

## EVALUATION OF SKILLS

Critical to the success of any program of teaching communication is the evaluation process.^45^ It is no secret that medical students, like their student colleagues elsewhere, are motivated strongly by the evaluation process. Unless there is a serious evaluation process at each portion of the course there will be a tendency to slight the course. Students who do not demonstrate acceptable performance skills should receive remedial training until they achieve the appropriate level of performance. The introduction of the clinical skills part to the United States Medical Licensing Examination (USMLE) process in 2004, with a major component consisting of communication skill evaluation, has been very important in providing a message to medical students and physicians that these skills are essential *sine qua non* for practicing medicine.

## IMPACT OF COMPUTERIZATION

There have been a number of recent changes in the practice of medicine that have complicated the issue of communication still further and that require specific attention. The increasing use of the computerized medical record with the computer on the physician’s desk has introduced an “intruder” to complicate further the physician–patient relationship and the communication between physician and patient. The use of the computerized medical record has many advantages and is clearly here to stay. But all too frequently we now hear patient complaints about the physician who seems to have more eye contact with the computer than with the patient whom he/she is treating. Special training must be included to prepare the physician to cope with this “intruder”.^46^

The handling of physician fatigue and the antecedent associated errors by shortening house officer duty hours have introduced another source of errors – those involved in the “hand-off procedure^47^ which occurs so much more frequently now. Communication between physician and physician is just as important a skill, which needs training, as that between physician and patient. In addition, as medicine becomes more complicated and as more different professionals are involved in patient care, it behooves physicians to improve their skills in communication with other essential members of the health care team. The era of the solo physician is long gone, and success in patient management depends in no small degree on excellent relationships and effective respectful communication with other health care professionals.

While most of the emphasis on communication teaching in medical schools focuses appropriately on the one-on-one contact between physician and patient, it is clear that there are other important aspects of communication which are an essential part of a physician’s role; these include contacts with families, administrators, students, news media, and the public at large. These areas too deserve attention.

## References

[1] Simpson M, Buckman R, Stewart M (1991). Doctor-patient-communication: The Toronto consensus statement. BMJ.

[2] Education Committee of the General Medical Council (1993). Tomorrow's doctors. Recommendations on Undergraduate Medical Education.

[3] Association of American Medical Colleges (1998). Learning Objectives for Medical Student Education. Guidelines for Medical Schools.

[4] (1992). Consensus statement from the workshop on the teaching and assessment of communication skills in Canadian medical schools. CMAJ.

[5] Makoul G, Schofield T (1999). Communication teaching and assessment in medical education: an international consensus statement. Patient EducCouns.

[6] Beeson PB (1980). Changes in medical therapy during the past half century. Medicine (Baltimore).

[7] (2008). Doctors and patients now at odds.

[8] Gibbs N (1989). Sick and tired. Time Magazine.

[9] Levinson W, Roter DL, Mullooly JP, Dull VT, Frankel RM (1997). Physician-patient communication. The relationship with malpractice claims among primary care physicians and surgeons. JAMA.

[10] Korsch BM, Gozzi EK, Francis V (1968). Gaps in doctor-patient communication I: Doctor–patient interaction and patient satisfaction. Pediatrics.

[11] Francis V, Korsch BM, Morris MJ (1969). Gaps in doctor-patient communication: patients' response to medical advice. N Engl J Med.

[12] Levinson W, Gorarara-Bhat R, Lamb J (2000). A study of patient clues and physician responses in primary care and surgical settings. JAMA.

[13] Trafford A (1995). The empathy gap. Washington Post.

[14] Hafferty F, Franks R (1994). The hidden curriculum, ethics teaching, and the structure of medical education. Acad Med.

[15] Hickson GB, Clayton EW, Entman SS (1994). Obstetricians' prior malpractice experience and patient's satisfaction with care. JAMA.

[16] Ambady N, Laplante D, Nguyen T, Rosenthal R, Chaumeton N, Levinson W (2002). Surgeons' voice: A clue to malpractice history. Surgery.

[17] Laine C, Davidoff F (1996). Patient-centered medicine: a professional evolution. JAMA.

[18] Peabody FW (1927). The care of the patient. JAMA.

[19] Engel GL (1977). The need for a new medical model: A challenge for biomedicine. Science.

[20] Engel GL (1977). The care of the patient: Art or science?. Johns Hopkins Med J.

[21] Maguire P, Fairbairn S, Fletcher C (1986). Consultation skill of young doctors II: Most young doctors are bad at giving information. BMJ.

[22] Becker M, Maiman L (1975). Sociobehavioral determinants of compliance with health and medical care recommendations. Med Care.

[23] Beckman HB, Frankel RM (1984). The effect of physician behavior on the collection of data. Ann Intern Med.

[24] Stewart MA (1995). Effective physician-patient communication and health outcomes: a review. CMAJ.

[25] Clark NM, Cabana MD, Nan B (2008). The clinician-patient partnership paradigm: outcomes associated with physician communication behavior. Clin Pediatr (Phila).

[26] (1986). Predictors of outcome in headache patients presenting to family physicians – A one year prospective study. Headache Study Group of the University of Western Ontario. Headache.

[27] Association of American Medical Colleges (1999). Medical School Objectives Project – Report III; Contemporary Issues in Medicine: Communication in Medicine.

[28] Losh DP, Mauksch LB, Arnold RW (2005). Teaching inpatient communication skills to medical students: an innovative strategy. Acad Med.

[29] Van Dalen J, Zuidweg J, Collet J (1989). The curriculum of communication skills teaching at Maastricht Medical School. Med Educ.

[30] Novack DH, Dube C, Goldstein MG (1992). Teaching medical interviewing: a basic course on interviewing and the physician-patient relationship. Arch Intern Med.

[31] Merton RK, Reader GG, Kendall PL (1957). The Student-Physician: Introductory Studies in the Sociology of Medical Education.

[32] Becker HS (1961). Boys in White: Student Culture in Medical School.

[33] Hafferty FW (1991). Into the Valley: Death and the Socialization of Medical Students.

[34] Newton BW, Barber L, Clardy J, Cleveland E, O'Sullivan P (2008). Is there hardening of the heart during medical school?. Acad Med.

[35] Hojat M, Vergare MJ, Maxwell K (2009). The devil is in the third year: A longitudinal study of erosion of empathy in medical school. Acad Med.

[36] Kripulani S, Bussey-Jones J, Katz MG, Genao I (2006). A prescription for cultural competence in medical education. J Gen Intern Med.

[37] Fadiman A (1998). The Spirit Catches You and You Fall Down.

[38] Galil A, Glick S, Flusser H (1996). Teaching medical students about disability: community-based approach. Medical Teacher.

[39] Alroy G, Ber R (1982). Doctor-patient relationship and the medical student: the use of trigger films. J Med Educ.

[40] Menahem S (1988). Trigger segments: towards improving listening skills. Med Educ.

[41] Rabin D, Rabin PL, Rabin R (1983). Compounding the ordeal of ALS: Isolation from my fellow physicians. N Engl J Med.

[42] Viner ED (1985). Life at the other end of the endotracheal tube: A physician's personal view of critical illness. Prog Crit Care Med.

[43] Lear MW (1980). Heartsounds: The Story of a Love and Loss.

[44] Margalit AP, Glick SM, Benbassat J, Cohen A, Katz M (2005). Promoting a biopsychosocial orientation in family practice. Med Teach.

[45] Boon H, Stewart M (1998). Patient-physician communication assessment instruments: 1986 to 1996 in review. Patient Educ Couns.

[46] Shachak A, Reis S (2009). The impact of electronic medical records on patient-doctor communication during consultation: a narrative literature review. J Eval Clin Pract.

[47] Solet DJ, Norvell JM, Rutan GH, Frankel RM (2005). Lost in translation: challenges and opportunities in physician-to-physician communication during patient handoffs. Acad Med.

